# An unconditional distribution learning advantage with shallow quantum circuits

**DOI:** 10.1038/s41534-026-01370-4

**Published:** 2026-11-10

**Authors:** N. Pirnay, S. Jerbi, J.-P. Seifert, J. Eisert

**Affiliations:** 1 Electrical Engineering and Computer Science, Technische Universität Berlin, Berlin, Germany; 2 Dahlem Center for Complex Quantum Systems, Freie Universität Berlin, Berlin, Germany; 3 Fraunhofer Heinrich Hertz Institute, Berlin, Germany; 4 Helmholtz-Zentrum Berlin für Materialien und Energie, Berlin, Germany

**Keywords:** Engineering, Mathematics and computing, Physics

## Abstract

One of the core challenges of research in quantum computing is concerned with the question whether quantum advantages can be found for near-term quantum circuits that have implications for practical applications. Motivated by this mindset, in this work, we prove an unconditional quantum advantage in the *probably approximately correct* (PAC) distribution learning framework with shallow quantum circuit hypotheses. We identify a meaningful generative distribution learning problem where constant-depth quantum circuits using one and two qubit gates are superior compared to constant-depth bounded fan-in and fan-out classical circuits as a choice for hypothesis classes. We hence prove a PAC distribution learning separation for shallow quantum circuits over shallow classical circuits. We do so by building on recent results by Bene Watts and Parham on unconditional quantum advantages for sampling tasks with shallow circuits, which we technically uplift to a hyperplane learning problem, identifying non-local correlations as the origin of the quantum advantage.

## Introduction

Machine learning has changed the world we live in: It is relevant for most algorithms that make predictions based on past training data. With quantum algorithms having been proven to provide super-polynomial advantages for certain highly structured^1,2^ or largely paradigmatic sampling^3,4^ problems, it is an extremely natural question to ask to what extent near-term quantum computers may assist in tackling machine learning tasks that have a practical inclination. This is a core theme of the emergent field of quantum machine learning^5,6^. Identifying such advantages is, however, much less of a straightforward task than one might think: Quantum computers are known to be good at addressing highly structured problems, while machine learning algorithms are generalizing from samples^7^. Also, such advantages of quantum over classical algorithms could come in the flavor of advantages in sample complexity, in computational complexity, or in generalization.

For some highly structured data, it has been shown that quantum computers indeed offer such super-polynomial advantages in computational complexity for meaningful and well-defined learning tasks with classical data^8^. This is true in particular for *probably approximately correct* (PAC) generator^9^ and density modeling^10^ as well as for classification tasks^11^ and reinforcement learning^12^. These quantum algorithms provide fair and sound advantages over all possible classical algorithms that can be proven under plausible and mild technical assumptions. It is worth noting that these advantages also do not suffer from hidden bottlenecks of quantum data preparation—an issue raised for prior works^13^. These insights are motivating for the field, in that they show that one can expect advantages of this type to be possible. That said, they only work for data that are precisely engineered and not very plausible from a practical perspective. Above all, they resort to full fault-tolerant quantum computers, which to date are still fictitious machines.

For this reason, one of the core questions of the field—if not the most important one at the present stage—is to find out whether shallow quantum circuits may offer quantum advantages that are plausible for near-term quantum architectures. The little we know about this important question to date lets us take a rather pessimistic viewpoint^14^. The need for such results is aggravated by the observation that in the absence of quantum error correction, there is increasing evidence that all one has are logarithmically deep quantum circuits before the effect of quantum noise sets in refs. ^15,16^, rendering potential quantum advantages void. So, can we hope for short quantum circuits to offer advantages in quantum machine learning?

In this work, we comprehensively address this question, taking steps towards finding practically minded quantum advantages for shallow quantum circuits. We do so by strongly building on the results of ref. ^17^ that show unconditional quantum advantages for evaluating certain functions and for sampling tasks which in turn build on ref. ^18^. The quantum learnability of constant-depth classical circuits has also been considered in ref. ^19^. Such studies are here brought to a new level in the distribution learning context, by adding a new technical twist: We introduce a hyperplane learning problem that allows to uplift the sampling problem at hand to a meaningful PAC distribution learning problem.

Specifically, we formulate a class of generative PAC distribution learning problems, where we are given examples from some unknown distribution in a distribution class. The goal is to output a generator for that distribution, that is, some hypothesis exposing an output distribution which is close to the unknown target distribution. We show that we can learn a constant-depth quantum circuit, consisting of one- and two-qubit gates, which achieves a level of accuracy arbitrarily close to the target distribution. In contrast, any classical circuit with constant depth and bounded fan-in gates is shown to achieve less proximity to the target distribution. Thus, showing an advantage of QNC^0^ over NC^0^ as hypothesis classes for these distributions.

Ultimately, the quantum advantage we demonstrate relies crucially on *non-local correlations* present in certain entangled states. These correlations enable the circuit to represent global features in shallow depth, in contrast to NC^0^ circuits whose local connectivity prevents similar representations.

The state prepared is not quite a GHZ state, as this is not within reach for constant depth quantum circuits^20^, as the GHZ state qualifies as being a “topological state”^21^. But with quantum circuits assisted by measurements and conditioned quantum dynamics, this is possible. Instead, what is called a “binary tree poor man’s GHZ state” is being prepared. Seen in this way, our work is also interesting from the perspective of the role of *measurements* in quantum computing^22^—a question that is presently much discussed in research on quantum computing and condensed matter physics: While we do not make use of mid-circuit measurements, in the preparation of the above state, measurements still play an important role in the preparation.

While our work builds on and makes use of the results of ref. ^17^, it is important to distinguish between which elements are inherited and which constitute genuine technical innovations. Reference ^17^ establishes a sampling separation for a single family of distributions {*D*_*n*_} for which constant-depth quantum circuits can sample more accurately than constant-depth classical circuits. Our technical contribution is to lift this sampling separation into a genuine learning-theoretic setting. First, we introduce a distribution class ${{{\mathcal{D}}}}=\{{D}_{n,p,s}\}$ indexed by a hidden shift parameter *s*, thereby defining a non-trivial family of learning tasks rather than a single distribution per problem size. Second, we provide an explicit PAC generator learning algorithm that recovers the hidden parameter *s* from samples and outputs a constant-depth quantum circuit generating the target distribution. Third, by combining this learner with the classical sampling lower bound inherited from ref. ^17^, we obtain a separation between QNC^0^ and NC^0^ as hypothesis classes in the PAC generator learning framework. Finally, on a more conceptual level, our intention has been to bring quantum advantages for sampling problems resorting to relatively shallow quantum circuits closer to applications with a practical flavor, here with a connotation with machine learning.

## Results

### Preliminaries

Throughout this work, we will be concerned with tasks of learning probability distributions, as is common in mathematical learning theory. We begin by giving the definition for a *generator* of a distribution.

#### Definition 1

(Generator for *D*) Let *D* be a discrete probability distribution over {0, 1}^*n*^ and *U* be the uniform distribution. A generator for *D* is any function GEN_*D*_: {0, 1}^*m*^ → {0, 1}^*n*^ that on uniformly random inputs outputs samples according to *D*, i.e.,

1 $${\Pr }_{x \sim U({\{0,1\}}^{m})}\left[{{{{\rm{GEN}}}}}_{D}(x)=y\right]=D(y).$$

*We sometimes also write* GEN_*D*_(*U*): = *D*.

In this work, we are primarily interested in two classes of generators: QNC^0^, which are constant-depth *quantum* circuits that consist of one- and two-qubit gates, and NC^0^, which are constant-depth *classical* circuits with bounded fan-in gates. Let us now define *PAC learning* of a generator.

#### Definition 2

((*ϵ*, *δ*)-PAC generator learner for ${{{\mathcal{D}}}}$) Let ${{{\mathcal{D}}}}$ be a class of discrete probability distributions over {0, 1}^*n*^. Given some fixed *ϵ*, *δ* ∈ (0, 1) an algorithm ${{{\mathcal{A}}}}$ is an (*ϵ*, *δ*)-PAC generator (GEN) learner of ${{{\mathcal{D}}}}$, if for all $D\in {{{\mathcal{D}}}}$, when given access to samples from *D*, with probability at least 1 − *δ*, ${{{\mathcal{A}}}}$ outputs a generator for some distribution $D^{\prime}$, satisfying

2 $$\begin{array}{r}{d}_{TV}(D,{D}^{{\prime} })\le \epsilon ,\end{array}$$

where *d*_*TV*_ is the total variation distance. We call ${{{\mathcal{A}}}}$ efficient if its runtime (and sample) complexity is *O*(poly(*n*)). We say that ${{{\mathcal{D}}}}$ is (*ϵ*, *δ*)-PAC generator learnable with ${{{\mathcal{H}}}}$, if an efficient ${{{\mathcal{A}}}}$ exists and if we constrain the generator to some generator class ${{{\mathcal{H}}}}$.

In this work, we construct a class of distributions that are PAC generator-learnable with arbitrarily low error *ϵ* and failure probability *δ*, if the generator is chosen from QNC^0^. If, however, the generator is chosen from NC^0^, such *ϵ*, *δ* are not achievable.

### From sampling advantage to learning advantage

The learning separation proven here derives from a separation in a sampling problem. Specifically, we build on a previously established family of distributions {*D*_*n*_} for which, asymptotically in *n*, constant-depth quantum circuits can sample with better accuracy than constant-depth classical circuits. For convenience, we restate the sampling statement that serves as the starting point of our construction^17^.

#### Theorem 3

(Inherited sampling separation, adapted from ref. ^17^) For each *δ* ∈ (0, 1), there exists a family of distributions $\left\{{D}_{n}\right\}$ such that for each $n\in {\mathbb{N}},{D}_{n}$ is a distribution over {0, 1}^*n*^ and

1. There exists a constant-depth quantum circuit which takes state vector $\left\vert {0}^{n}\right\rangle$ as input and produces a distribution which has total variation distance at most $\frac{1}{6}+O\left({n}^{-c}\right)$ from *D*_*n*_ for some *c* ∈ (0, 1).
2. Each classical circuit with fan-in 2 which takes *n* + *n*^*δ*^ random bits as input and whose output distribution has total variation distance at most $\frac{1}{2}-\omega (1/\log n)$ from *D*_*n*_ has depth $\Omega (\log \log n)$.

**Fig. 1:**
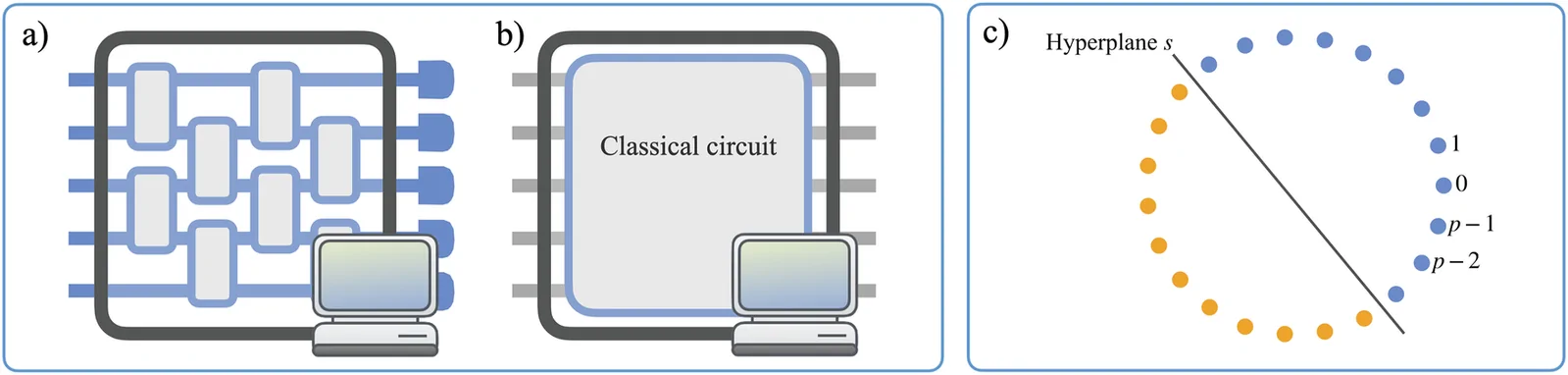
Shallow-depth separations. **a** Constant depth quantum circuits with one- and two-qubit gates are compared with **b** constant-depth bounded fan-in classical circuits. **c** The majmod_*p*,*s*_(*x*) function acting on the finite field ${{\mathbb{F}}}_{p}$. In this case, *p* = 23 and *s* = 3. The points are elements in ${{\mathbb{F}}}_{p}$ and are blue or orange if majmod_*p*,*s*_(*x*) = 0 or 1, respectively.

The statement above provides an unconditional *sampling* advantage of QNC^0^ over NC^0^. Our goal, however, is to obtain a PAC generator *learning* advantage rather than a separation for a fixed sampling task. A direct but uninteresting way to turn Theorem 3 into a learning problem would be to define the target class to contain only one distribution *D*_*n*_ for each problem size *n*. In that case, however, there is essentially no latent structure to infer from samples, so the resulting task would not capture the spirit of learning. To obtain a genuine learning problem, we introduce a hidden parameter into the underlying majority-mod-*p* structure. Concretely, we promote the construction to a family indexed by a shift $s\in {{\mathbb{F}}}_{p}$, so that learning amounts to identifying this hidden parameter from samples. This leads to a natural hyperplane-learning variant of the majority-mod-*p* function, which we call “majority mod *p* under hyperplane *s*”. That is, we define

3 $${{\mbox{majmod}}}_{p,s}(x)=\left\{\begin{array}{ll}0,\,{{\mbox{if}}}\,| x| +s < p/2\quad{{{\mathrm{mod}}}}\,\,\,p,\\ 1,\,{{\mbox{if}}}\,| x| +s > p/2\quad{{{\mathrm{mod}}}}\,\,\,p,\end{array}\right.$$

for each *x* ∈ {0, 1}^*n*−1^, prime *p* and $s\in {{\mathbb{F}}}_{p}$, where ${{\mathbb{F}}}_{p}$ denotes the standard finite field of order *p*. We will occasionally abuse the notation and allow majmod_*p*,*s*_ to take an *integer* argument that replaces ∣*x*∣ in the calculation of the function, i.e., majmod_*p*,*s*_(*x*) = majmod_*p*,*s*_(∣*x*∣). Figure 1 shows the behavior of the majmod_*p*,*s*_ function. Using this shifted majority-mod-*p* function, consider the distribution

4 $${D}_{n}:={(x,{{{{\rm{majmod}}}}}_{p,s}(x)\oplus {{{\rm{parity}}}}(x))}_{x \sim U}.$$

Now, if we want to learn a generator for *D*_*n*_, when *n*, *p* are given, one needs to learn the hyperplane *s* from *m* examples of *D*_*n*_. At an intuitive level, this already has the form of a learning problem: the input examples are sampled from a family indexed by an unknown parameter, and the learner must infer that parameter well enough to output a matching generator. Moreover, the inherited sampling separation strongly suggests an asymmetry between shallow quantum and shallow classical hypotheses for this task: shallow classical circuits remain limited by the same locality obstruction, whereas shallow quantum circuits can exploit the corresponding global correlations. However, this first reformulation would still inherit a key limitation of Theorem 3: the quantum generator would only be guaranteed to approximate the target distribution up to a fixed constant error (at least $\frac{1}{6}$), rather than to arbitrary accuracy.

Conversely, in the PAC learning setting, we require that for some probability of failure *δ*, we can make the error *ϵ* arbitrarily low (usually by taking more examples). Inspired by these observations, we will, in the subsequent sections, introduce a new target distribution class, for which we show that we can learn constant-depth quantum generators that generate the target distributions (with arbitrary precision). At the same time, we show that any constant-depth classical generator cannot approximate the target distributions up to some constant error.

### PAC generator learning advantage of QNC^0^ over NC^0^

To obtain a PAC learning advantage with QNC^0^ with arbitrary precision, we proceed in three steps. First, we define the target distribution class and explain its construction. Second, we give a PAC generator learning algorithm for this class using constant-depth quantum circuits. Third, we show that any PAC generator learning algorithm for that distribution class that uses constant-depth classical circuits must make some non-negligible error.

### The target distribution class ${{{\mathcal{D}}}}$

We will proceed in two steps. First, we will define a distribution of the form (*Z*, pmmajmod_*p*,*s*_(*Z*)), where pmmajmod_*p*,*s*_ is to be defined. In the second step, we give a constant-depth quantum circuit that approximately samples from the (*Z*, pmmajmod_*p*,*s*_(*Z*)) distribution. We then define the Born distribution of that constant-depth quantum circuit to be the target distribution of our PAC learning task.

**Fig. 2:**
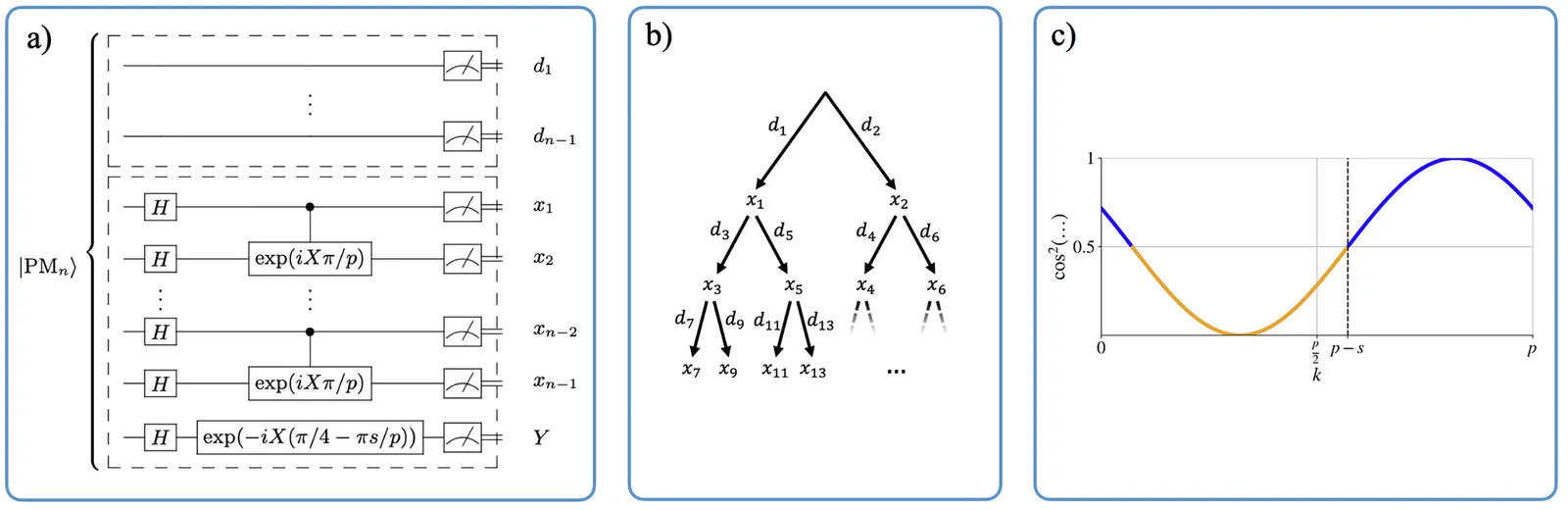
A separating distribution. **a** Unitary circuit producing the target distribution *D*_*n*,*p*,*s*_. The upper box indicates the *n* − 1 “edge” qubits of the state vector $\vert {{{{\rm{PM}}}}}_{n}\rangle$. The lower box indicates the *n* “vertex” qubits of the same state, where *Y* is the root qubit. Note that the input state vector $\vert {{{{\rm{PM}}}}}_{n}\rangle$ can be prepared in constant-depth, following Lemma 4. The gates ${U}_{m,\frac{\pi }{p}}$ and *C*_*m*_ act on blocks of $m=\lceil2/c+1\rceil$ qubits, for an arbitrary constant *c* ∈ (0, 1/3), and are defined in Section A of the Supplementary Information. **b** Balanced binary tree, where the vertices and edges are assigned to the binary variables *x*_1_, …, *x*_*n*−1_ and *d*_1_, …, *d*_*n*−1_, respectively, useful for the definition of the state vector $\vert {{{{\rm{PM}}}}}_{n}\rangle$. **c** Visualization of ${\cos }^{2}(-\frac{\pi }{4}+\frac{\pi }{p}(k+s))$, a core function in the definition of the target distribution *D*_*n*,*p*,*s*_. The blue and orange portions of the plot signal the majmod_*p*,*s*_(*k*) = 0 and 1 values, respectively, as in Fig. 1c.

We define the distribution (*Z*, pmmajmod_*p*,*s*_(*Z*)) constructively. For that, let us first consider the balanced binary tree in Fig. 2b. Call its vertex variables *x*_1_, …, *x*_*n*−1_ ∈ {0, 1} and its edge variables *d*_1_, …, *d*_*n*−1_ ∈ {0, 1}. Now define the function h(*d*): {0, 1}^*n*−1^ → {0, 1}^*n*−1^ by

5 $${{{\rm{h}}}}{(d)}_{i}= {\bigoplus}_{j:{d}_{j}\in P({x}_{i})}{d}_{j}\,i\in \{1,2,\ldots ,n-1\},$$

where *P*(*x*_*i*_) is the set of edges contained in the (unique) path going from the root to *x*_*i*_. Essentially, h(*d*)_*i*_ is the parity of the edge variables along the path from the root to *x*_*i*_. Given this function, we can define the so-called *binary tree poor man’s GHZ state* vector as

6 $$\left\vert {{{{\rm{PM}}}}}_{n}\right\rangle = 	 \sum\limits_{d\in {\{0,1\}}^{n-1}}\frac{1}{{2}^{(n-1)/2}}\left\vert d\right\rangle \\ \quad 	 \otimes \frac{1}{\sqrt{2}}\left(\left\vert {{{\rm{h}}}}(d)0\right\rangle +\left\vert \overline{{{{\rm{h}}}}(d)}1\right\rangle \right),$$

where we call the first *n* − 1 qubits of $\left\vert {{{{\rm{PM}}}}}_{n}\right\rangle$ “edge” qubits, and the following *n* qubits “vertex” qubits. Note that the rightmost qubit corresponds to the root vertex, whose value is written explicitly. The state vector $\left\vert {{{{\rm{PM}}}}}_{n}\right\rangle$ is a special case of the *poor man’s cat state vector*^20^, specialized here to the balanced binary tree. Its role is to supply long-range correlations, and it can be prepared with a constant-depth unitary quantum circuit^17,20^. The “poor man’s” attribute comes from the fact that the state can be created with little resources (i.e., in constant depth) but, compared to the GHZ state vector $\frac{1}{\sqrt{2}}({\left\vert 0\right\rangle }^{\otimes n}+{\left\vert 1\right\rangle }^{\otimes n})$, has random bit flips that cannot be corrected in constant depth.

#### Theorem 4

(Constant-depth preparation of $\left\vert {{{{\rm{PM}}}}}_{n}\right\rangle$^17^) For any *n*, the state vector $\left\vert {{{{\rm{PM}}}}}_{n}\right\rangle$ can be constructed by a depth-3 quantum circuit consisting of 1 and 2 qubit gates acting on 2*n* − 1 qubits.

Intuitively, the importance of Lemma 4 is that it provides a shallow quantum mechanism for encoding global information about many bits into correlations with the final output label. This is the structural ingredient that will ultimately separate the quantum and classical hypothesis classes.

Now, let us consider the distribution that takes the form (*Z*, pmmajmod_*p*,*s*_(*Z*)), where *Z* is a uniformly sampled (2*n* − 2)-bit string and pmmajmod_*p*,*s*_ is what we call the “poor man’s majority mod *p*” function, defined as

7 $$\begin{array}{rcl}&&{{\mbox{pmmajmod}}}_{p,s}:{\{0,1\}}^{n-1}\times {\{0,1\}}^{n-1}\to \{0,1\}\\ &&{{\mbox{pmmajmod}}}_{p,s}(Z)={{\mbox{pmmajmod}}}_{p,s}(d,x)\\ &&:={{\mbox{majmod}}}_{p,s}\left({\sum }_{i = 0}^{n-1}{x}_{i}{(-1)}^{{{{\rm{h}}}}}{(d)}_{i}\right)\oplus {{{\rm{parity}}}}(x).\end{array}$$

The next step is to relate this structured distribution to a shallow quantum circuit. The relation is inherited from the underlying shallow-circuit construction^17^ and extends directly to the shifted function pmmajmod_*p*,*s*_. This yields the following approximation result.

#### Theorem 5

(Constant-depth approximation of the shifted target distribution) For *n* sufficiently large and prime *p* = *n*^*c*^ for any constant *c* ∈ (0, 1/2) there exists a constant-depth quantum circuit consisting of one- and two-qubit unitary gates that takes the (2*n* − 1)-qubit all zeros state as input and produces an output which, when measured in the computational basis, samples from a (2*n* − 1)-bit distribution $({Z}^{{\prime} },Y)$ which correlates approximately with the distribution (*Z*, pmmajmod_*p*,*s*_(*Z*)),

8 $$\begin{array}{rcl}&&{d}_{TV}(({Z}^{{\prime} },Y),(Z,{{\mbox{pmmajmod}}}_{p,s}(Z)))\\ &&\le \frac{1}{2}-\frac{1}{\pi }+O({n}^{-c}).\end{array}$$

The quantum circuit to generate this approximate distribution $({Z}^{{\prime} },Y)$ is given in Fig. 2a. The Born distribution $({Z}^{{\prime} },Y)$ generated by this circuit is our target distribution.

To get a sense of why Lemma 5 holds, note that the state vector of the last qubit in Fig. 2a, after measuring the first 2*n* − 2 qubits in the state vector $\left\vert d,x\right\rangle$, is *approximately* given by

9 $$\begin{array}{rcl}\left\vert \psi \right\rangle &\approx &\cos \left(-\frac{\pi }{4}+\frac{\pi }{p}\left({\sum }_{i=0}^{n-1}{x}_{i}{(-1)}^{h{(d)}_{i}}+s\right)\right)\\ &\times &\left\vert \overline{{{{\rm{parity}}}}(x)}\right\rangle \hfill\\ &+&\sin \left(-\frac{\pi }{4}+\frac{\pi }{p}\left({\sum }_{i=0}^{n-1}{x}_{i}{(-1)}^{h{(d)}_{i}}+s\right)\right)\\ &\times &\left\vert \overline{{{{\rm{parity}}}}(x)}\right\rangle .\hfill\end{array}$$

And from Fig. 2c, it can be seen that, to good approximation, ${\cos }^{2}(-\frac{\pi }{4}+\frac{\pi }{p}(k+s))$ is inversely correlated with majmod_*p*,*s*_(*k*). This is the desired property to approximate (*Z*, pmmajmod_*p*,*s*_(*Z*)) as we want the last bit to equal $\overline{(x)}$ when majmod_*p*,*s*_(*k*) = 0 and $\overline{{{{\rm{parity}}}}(x)}$ otherwise.

Now, we are in a position to define our *target distribution class* as

10 $${{{\mathcal{D}}}}:=\{{D}_{n,p,s}| n\in {\mathbb{N}}\,{\mbox{, prime}}\,p\in {{\mathbb{Z}}}^{+}\,{\mbox{,}}\,s\in {{\mathbb{F}}}_{p}\}.$$

The distribution *D*_*n*,*p*,*s*_ is given by the Born distribution of the unitary constant-depth quantum circuit in Fig. 2a. A direct corollary of this and Lemma 4 is the following statement.

#### Corollary 6

(Optimal quantum circuit) The optimal quantum circuit to generate the distribution *D*_*n*,*p*,*s*_ is at most constant-depth.

### PAC generator learning algorithm for ${{{\mathcal{D}}}}$ with QNC^0^

We now proceed by presenting an explicit learning algorithm that learns *s* from examples of *D*_*n*,*p*,*s*_. From the knowledge of *s*, one can then trivially construct a generator for *D*_*n*,*p*,*s*_ in QNC^0^, by simply using the quantum circuit in Fig. 2a.

#### Theorem 7

(PAC generator learning algorithm of the quantum distribution) There exists a polynomial-time PAC generator learning algorithm for ${{{\mathcal{D}}}}$, that for sufficiently large prime *p* ∈ *O*(*n*^1/3^), any *δ* > 0, and any $s\in {{\mathbb{F}}}_{p}$, knowing *n* and *p*, outputs a description of a constant-depth quantum circuit whose Born distribution $D{\prime}$ satisfies

11 $${d}_{TV}({D}_{n,p,s},{D}^{{\prime} })=0$$

with probability 1 − *δ*, and which uses $M\,\in \,O\left({p}^{4}\log \left(\frac{p}{\delta }\right)\right)$ examples of *D*_*n*,*p*,*s*_.

The full proof can be found in Section B of the Supplementary Information, but we give a high-level overview of the ideas. We are given *M* examples from the discrete distribution *D*_*n*,*p*,*s*_ and *n*, *p*. From Eq. (9), we know that under some approximate distribution *P*, conditioned on a given value of (*d*, *x*), the probability to observe $(d,x,\overline{(x)})$ is $f(k)={\cos }^{2}(-\frac{\pi }{4}+\frac{\pi }{p}\left(k+s\right))$ for

12 $$k={\sum }_{i=0}^{n-1}{x}_{i}{(-1)}^{h{(d)}_{i}}\,{{{\mathrm{mod}}}}\,\,p.$$

The strategy for the learning algorithm is essentially to estimate *f*(*k*), for *k* = 1, …, *p*, from the *M* examples drawn from *D*_*n*,*p*,*s*_. Then, as depicted in Fig. 2c, we can identify ${k}^{* }=p-s\,{{\mathrm{mod}}}\,\,p$ as the coordinate for which *f*(k^*^) = 1/2 and *f*(k^*^ + 1) > 1/2. This way, we recover $s=p-{k}^{* }\,{{\mathrm{mod}}}\,\,p$. The only difficulty is in computing the values *f*(*k*) to sufficient precision from examples. We show that $M=O\left({p}^{4}\log \left(\frac{p}{\delta }\right)\right)$ examples are sufficient to guarantee enough precision to identify *s* with probability 1 − *δ*. Then we can output the quantum circuit in Fig. 2a, as the generator in QNC^0^. Since this circuit is exactly the circuit that generated the target distribution, we obtain an error *ϵ* = 0.

### Classical hardness

To show the classical hardness of PAC generator learning ${{{\mathcal{D}}}}$ with NC^0^, it suffices to show that, even for one fixed value of *s*, the distributions *D*_*n*,*p*,*s*_ are not in NC^0^. We use the following inherited lower bound for the special case *s* = 0.

#### Theorem 8

(Classical lower bound for the target family^17^) For each *δ* < 1, there exists an *ϵ* > 0 such that for all sufficiently large even integer *N* = 2*n* − 2 and prime number *p* = Θ(*N*^*α*^) for *α* ∈ (*δ*/3, 1/3): Let *f*: {0, 1}^*ℓ*^ → {0, 1}^*N*+1^ be an ${(\epsilon \log N)}^{1/2}$-local function, with *ℓ* ≤ *N* + *N*^*δ*^. Then ${d}_{TV}(f(U),(Z,{{\mbox{pmmajmod}}}_{p,s = 0}(Z)))\ge 1/2-O(1/\log N)$.

For a *d*-local function, each output bit depends on at most *d* input bits. Intuitively, the theorem above states that there exist no constant-depth classical circuits that can asymptotically sample from the distribution (*Z*, pmmajmod_*p*,*s*=0_(*Z*)) up to good fidelity, as constant-depth circuits are *O*(1)-local. This is the classical obstruction that underlies the learning separation. At a technical level, the proof for the theorem above combines the framework of ref. ^23^ with the major modification of considering the “poor man’s majority mod *p*” function instead of the “majority mod *p*” function.

### Summary of results

We are now in a position to state our main result, which is a meaningful advantage of a shallow quantum circuit over instances of classical circuits in a well-defined learning task.

#### Theorem 9

(Advantage of shallow quantum hypotheses) For the problem of learning a constant-depth generator for the distribution class ${{{\mathcal{D}}}}$, the hypothesis choice of QNC^0^ over NC^0^ yields an advantage in total variation distance of at least

$$\frac{1}{\pi }-O\left(\frac{1}{\log n}\right).$$

#### Proof

Due to Lemma 5, we know that

13 $$\begin{array}{rcl}&&{d}_{TV}({D}_{n,p,s},(Z,{{\mbox{pmmajmod}}}_{p,s}(Z)))\\ &&\le \frac{1}{2}-\frac{1}{\pi }+O({n}^{-c})\end{array}$$

for some constant *c* ∈ (0, 1/3), and due to Lemma 8 we know that

14 $$\begin{array}{rcl}&&{d}_{TV}(f(U),(Z,{{\mbox{pmmajmod}}}_{p,s}(Z)))\\ &&\ge 1/2-O\left(\frac{1}{\log N}\right),\end{array}$$

where *f* is any ${(\epsilon \log N)}^{1/2}$-local function. It follows from the reverse triangle inequality that

15 $$\begin{array}{rcl}&&{d}_{TV}(f(U),{D}_{n,p,s})\hfill\\ &&\ge \frac{1}{\pi }-O({n}^{-c})-O\left(\frac{1}{\log N}\right)\\ &&\ge \frac{1}{\pi }-O\left(\frac{1}{\log n}\right).\hfill\end{array}$$

Since *D*_*n*,*p*,*s*_ is generated by the constant-depth quantum circuit in Fig. 2a, and we can learn that circuit from examples to perfect precision, due to Lemma 7, we obtain the advantage gap as in Eq. (15) for learning a generator for the target distribution class ${{{\mathcal{D}}}}$ using constant-depth quantum circuits instead of constant-depth classical circuits.           □

The advantage ultimately arises from a mismatch between the global correlations produced by a shallow quantum circuit and the locality constraints of shallow classical circuits. Shallow quantum circuits can prepare an entangled state whose measurement outcomes approximately follow a distribution of the form (*Z*, pmmajmod_*p*,*s*_(*Z*)), where *Z* is uniformly random and the final output bit depends on a global property of *Z*. In contrast, any constant-depth classical circuit with bounded fan-in implements functions with limited locality: each output bit depends on only a small subset of the input bits. Similar structures appear in quantum-classical separations involving shallow circuits^17,18,24,25^.

### Technical remarks on the separation

It is important to spell out a few technical remarks about the separation proved here. To start with, our construction relies on quantum circuits that use arbitrary single-qubit rotations. However, the advantage is not merely an artifact of arbitrary rotations: the advantage persists even when the classical circuits are granted biased input bits, which corresponds to allowing arbitrary single-qubit rotations on the quantum side^17^.

Another subtlety arises from the asymmetry in fan-out capabilities between NC^0^ and QNC^0^. Classical NC^0^ circuits are allowed unbounded fan-out, while QNC^0^ circuits, as we define them, do not include this capability. One might therefore argue that the two classes are incomparable. However, we stress that the separation shown here still holds if we restrict NC^0^ to have bounded fan-out, which puts both classes on more equal footing. Further, note that NC^0^ circuits with bounded fan-out can be trivially simulated by a QNC^0^ circuit that has access to constantly-many additional qubits. Then, to make the comparison fair, we could allow the classical circuits to also use constantly-many additional input bits. In fact, in the separation we showed, the advantage persists even when the classical circuit has access to *n*^*δ*^ extra input bits.

Thus, while QNC^0^ vs NC^0^, both restricted to bounded fan-in and fan-out gates and equipped with constantly-many additional input bits, would already be a fair comparison, our result establishes a stronger separation in which NC^0^ is allowed unbounded fan-out and additional *n*^*δ*^ input bits. At the same time, there may well exist distributions that can be generated by NC^0^ circuits with unbounded fan-out but not by QNC^0^ circuits with bounded fan-out. Our claim is therefore not that QNC^0^ dominates NC^0^ as a circuit class in full generality, but rather that for the specific distribution learning problem considered here, QNC^0^ is the better hypothesis class.

## Discussion

One of the core questions of research on quantum computing is concerned with the question of whether one can meaningfully identify a quantum advantage in near-term devices that relates to a problem that has a practical flavor. In this work, we have investigated a learning problem in which well-defined families of constant-depth quantum circuits feature an advantage over families of constant-depth classical circuits. This problem is a meaningful and mathematically well-defined machine learning problem in the PAC generator learning sense.

The distribution class we consider exposes a natural hyperplane learning problem, which is closer to a real learning setting than prior works on quantum advantages for distribution learning^9,10^, which leverage cryptographic constructions and Shor’s algorithm. Further, in those works, due to their constructions, only one training sample is needed to learn the distribution, while here the quality of the hypothesis depends naturally on the amount of samples observed, which again resembles a more practical learning setting. It is important to note that learning with constant-depth hypotheses is also regularly studied in classical learning theory^26^. In fact, the concept class studied in this work is polynomial in size (in the problem parameters), which is also a common setting for showing learning advantages in the classical PAC literature^27^. In this way, the separation proven here between quantum and classical learners naturally ties in with the literature on classical mathematical learning theory. While our work is phrased with the near-term quantum era in mind, we emphasize that our findings are primarily conceptual in nature, contributing to showing that short circuits can exhibit quantum advantages, and do not immediately imply feasibility on today’s noisy intermediate-scale quantum devices. Indeed, arbitrary rotations and ideal gate fidelity are assumed, which pose challenges in a number of practical implementations.

So, our work answers the question of whether quantum advantages in machine learning problems with short quantum circuits can be found in the affirmative. Here, we are resorting to constant-depth quantum circuits.

An interesting direction for future work is to quantify the precise robustness of the advantage under specific noise models or to identify error mitigation schemes that preserve it. Since the construction only involves constant-depth circuits, the scheme inherits a degree of robustness to noise that is typical for shallow circuit architectures. A detailed quantitative analysis of this robustness, however, lies beyond the scope of the present work. That said, for any near-term scheme, one should expect to maintain coherence only to poly-logarithmic depth, as a strict asymptotic statement. Indeed, there are known rigorous results on limitations of non-error-corrected quantum circuits that would set in at logarithmic or poly-logarithmic depth^15,16^. The present work, finding quantum advantages at constant depth, leaves a lot of room for optimism on the use of quantum algorithms in the medium term. To precisely find out how large the intermediate regime is until classical simulation methods set in, identifying a “sweet spot” for quantum advantages is one of the core research questions of near-term quantum computing.

That said, this result is a stepping stone in a bigger program towards achieving fault-tolerant application-scale quantum computers^28^. Ideally, one should be able to show advantages also over deeper classical circuits. Also, while the problem considered is a meaningful problem and has a practical machine learning flavor to it, it still makes use of highly structured data, similar to other work showing quantum advantages in the learning context^9–12^. That is to say, while the specific distribution class considered here is deliberately tailored to exhibit a separation, such constructions are consistent in the quantum learning literature. We therefore view the present result as an exploration of the boundaries of learnability in principle and as a stepping stone toward stronger and more realistic quantum learning advantages. Ideally, future work should extend such separations to less structured data and to comparisons against more powerful classical models. Steps have also been taken to deal with realistic and unstructured data^29^, but again, such algorithms will presumably eventually require a fault-tolerant quantum computer.

It is also worth noting that while being related, the question discussed here is different from the question of actually learning shallow quantum circuits^30,31^, even though it will be interesting to further flesh out the precise connections. More work needs to be done to bring such ideas closer to reality and the realm of unstructured data. It is the hope that the present work can serve as a further inspiration along these lines of thought.

## Methods

The methods developed and employed in this work have already been introduced throughout the Results section, where they are presented together with the corresponding findings. All details are provided in full rigor in the Supplemental Material.

### Quantum learning task

At a high level, we explain how the sampling advantage established in Theorem 3 can be converted into a learning advantage. To obtain a PAC learning advantage with QNC^0^ at arbitrary precision, we proceed in three steps. First, we define a distribution of the form (*Z*, pmmajmod_*p*,*s*_(*Z*)), where pmmajmod_*p*,*s*_ is defined in the main text. Second, we construct a constant-depth quantum circuit that approximately samples from the distribution (*Z*, pmmajmod_*p*,*s*_(*Z*)). Third, we define the Born distribution of this constant-depth quantum circuit as the target distribution of our PAC learning task. Theorems 4 and 5 summarize these steps. Finally, we construct an explicit learning algorithm that learns the hidden parameter *s* from examples drawn from *D*_*n*,*p*,*s*_.

### Classical hardness

In a last step, Theorem 8 establishes the classical hardness of PAC generator learning for ${{{\mathcal{D}}}}$ with NC^0^. Specifically, we prove that, even for a fixed value of *s*, the distributions *D*_*n*,*p*,*s*_ cannot be generated by NC^0^ circuits.

## Supplementary information

Supplementary Information

## Data Availability

Data sharing is not applicable to this article as no datasets were generated or analyzed during the current study.

## Code availability

No code has been created in this work.
