# Supplementary material for: An unconditional distribution learning advantage with shallow quantum circuits
Source: npj Quantum Inf. 2026 Nov 10;12(1):184. doi: 10.1038/s41534-026-01370-4 (PMC13754569; doi:10.1038/s41534-026-01370-4)
Supplement: Supplementary file 1 [file 41534_2026_1370_MOESM1_ESM.pdf]

Supplementary Information:
An unconditional distribution learning advantage with shallow quantum circuits

A. DEFINING THE $U_{m,\theta}$ AND C_m GATES

To fully understand the construction of the $U_{m,\theta}$ and C_m gates, we need to stride out a little bit. Firstly, let us consider a different gate, namely, the $A_{m,\theta}$ gate. The $A_{m,\theta}$ gate is defined by its action on the computational basis state vectors as

$$A_{m,\theta} |x_1, x_2, \ldots, x_m\rangle = \exp(i\theta x_m X) |x_1\rangle \otimes \exp(i\theta x_1 X) |x_2\rangle \otimes \ldots \otimes \exp(i\theta x_{m-1} X) |x_m\rangle . \tag{S1}$$

A constant-depth circuit built from the gates $A_{m,\theta}$ provides an intermediate approximation to the structured distribution $(Z, \mathrm{pmmajmod}_{p,s}(Z))$. The following statement follows from Lemma 10 and Theorem 31 of Ref. [1] and a trivial generalization to s.

Theorem 1 (Intermediate approximation using $A_{m,\theta}$ gates [1])**.** *For any $p \in \mathbb{Z}^+$ there is a constant-depth circuit consisting of one- and two-qubit unitary gates and $A^\dagger_{m,\theta}$ operations (for arbitrary $m \geq 1$) which takes the $(2n-1)$-qubit all-zero state as input and produces an output state, which when measured in the computational basis, produces a distribution P with samples of the form (Z', Y), such that*

$$d_{TV}(P, (Z, \mathit{pmmajmod}_{p,s}(Z))) \leq \frac{1}{2} - \frac{1}{\pi} + \frac{1}{2p} + O(p^{3/2} e^{-n/4p^2}). \tag{S2}$$

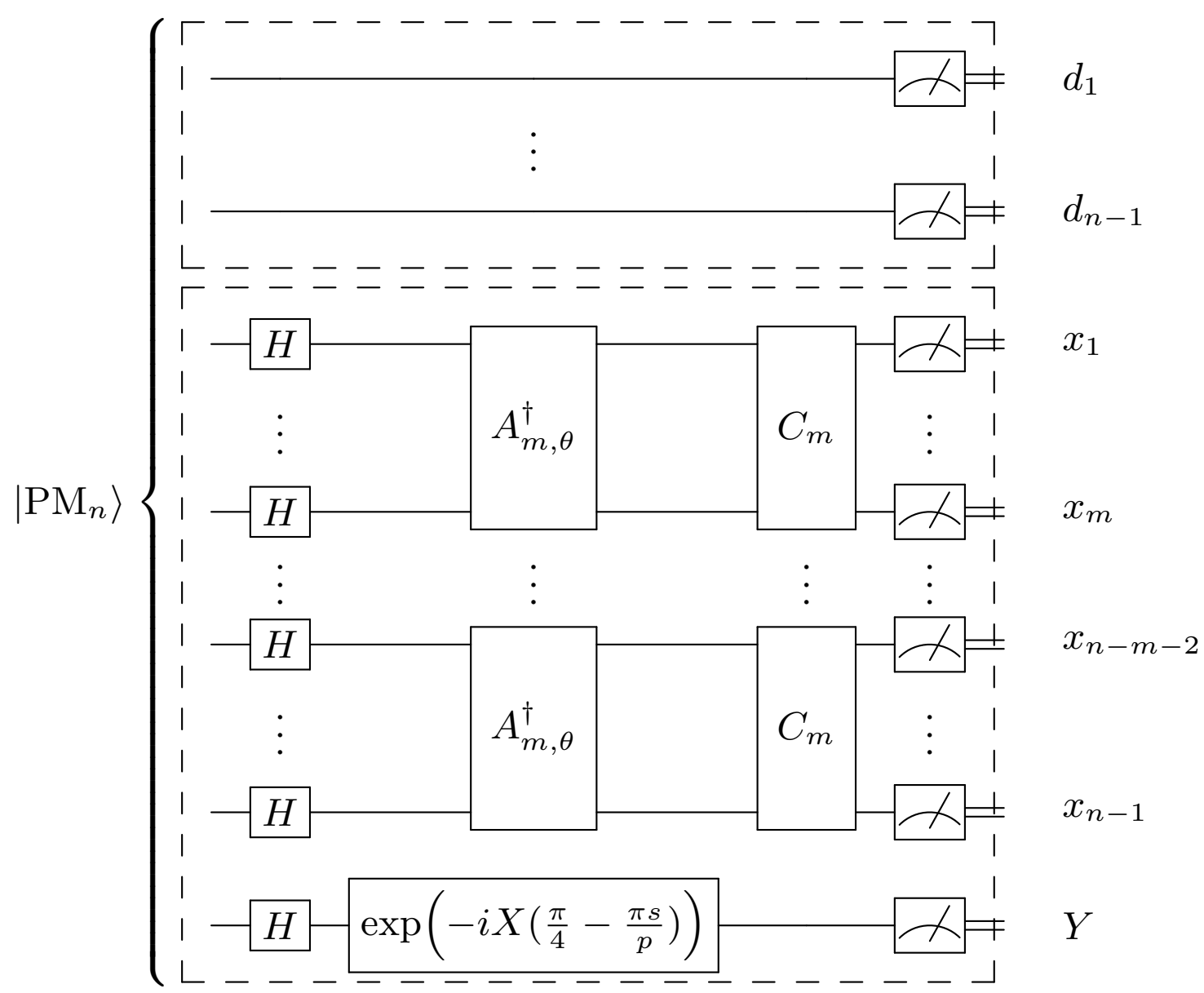

Figure S1. Constant-depth non-unitary circuit producing an approximate distribution P to $(Z, \mathrm{pmmajmod}_{p,s}(Z))$, for $\theta = \frac{\pi}{p}$. Note that the input state vector $|\mathrm{PM}_n\rangle$ can be prepared in constant-depth, following Theorem 4 in the main text.

The circuit that achieves this approximation is depicted in Fig. S1. The C_m gate denotes a permutation whose action on the m qubit computational basis state vector $|x_1, x_2, \ldots, x_m\rangle$ is given by

$$C_m |x_1, x_2, \ldots, x_m\rangle = |x_2, x_3, \ldots, x_m, x_1\rangle . \tag{S3}$$

The C_m gate can be implemented by a series of $m-1$ SWAP gates. Problematically, the $A_{m,\theta}$ gate is non-unitary. To solve this issue, the unitary gate $U_{m,\theta}$ is defined as

$$U_{m,\theta} := \begin{cases} A_{m,\theta} |x\rangle & \text{if } x \in B^m, \\ C^{-1}(A_{m,\theta} |x\rangle + i^{m+2|x|} \sin^m(\theta) A_{m,\theta} |\overline{x}\rangle) & \text{otherwise,} \end{cases} \tag{S4}$$

where

$$C := \sqrt{1 - \sin^{2m}(\theta)} \tag{S5}$$

is a normalizing constant and B^m is any set containing half the bit strings of length m with the property that for any $x \in \{0,1\}^m$ either $x \in B^m$ or $\overline{x} \in B^m$. Essentially, $U_{m,\theta}$ arises from applying the Gram-Schmidt orthonormalization to the problematic states output by $A_{m,\theta}$. It is shown that the unitary gate $U_{m,\theta}$ approximates the non-unitary gate $A_{m,\theta}$.

Lemma 2 (Unitary approximation of $A_{m,\theta}$ [1])**.** *For any m, there exists a unitary matrix $U_{m,\theta}$ satisfying*

$$||A_{m,\theta} - U_{m,\theta}||_F \in O(\theta^m) \tag{S6}$$

as $\theta \to 0$.

In particular, setting $\theta = \frac{\pi}{p}$, with $p = n^c$ for some constant $c \in (0, 1/2)$, the equations (116)-(119) in Ref. [1] give us the following.

Lemma 3 (Closeness of unitaries)**.** *For any m, and $\theta = \frac{\pi}{p}$, and $p = n^c$ for some constant $c \in (0, 1/2)$, there exists a unitary matrix $U_{m,\theta}$ satisfying*

$$||A_{m,\theta} - U_{m,\theta}||_\infty \in O(n^{-mc}). \tag{S7}$$

And thus Theorem 5 in the main text follows. We further call the distribution Q, which is produced by the circuit in Fig. 2.a) (main text) and uses the $U^\dagger_{m,\theta}$ gates. Now, let $m = \lceil 2/c + 1 \rceil$. Note that the circuits producing P and Q differ only by replacing every $A^\dagger_{m,\theta}$ gate with a $U^\dagger_{m,\theta}$ gate. There are $O(n)$ such gates. It follows from using Lemma 3 and following the steps of equations (118)-(121) in Ref. [1], that

$$d_{TV}(P, Q) \in O(n) \times O(n^{-mc}) = O(n^{-c-1}) = O\left(\frac{1}{np}\right). \tag{S8}$$

We can now turn to the actual circuit compilation of $U_{m,\theta}$. The $U_{m,\theta}$ gates can be compiled into arbitrary one-qubit gates and two-qubit CNOT gates [1]. Following the discussions in Ref. [2], any operator on m qubits can be performed with at most $O(m^3 4^m)$ two-qubit gates, using the methods described in Ref. [3] and in a runtime that is bounded by a function of m. Further, any of those two-qubit gates can be decomposed into a sequence of at most 5 one-qubit gates and CNOT gates, following the results of Ref. [4]. Thus, for constant m and any θ, there exists a constant time algorithm that compiles the $U_{m,\theta}$ gates into a constant-depth sequence of arbitrary one-qubit gates and CNOT gates. The algorithm to obtain the circuit compilation to any unitary operator is also excellently explained in Chapter 5.4 of Ref. [5].

B. LEARNING ALGORITHM

In this section, we present the detailed rigorous analysis of the learning algorithm used in the main text. We show that the circuits generating the distributions $Q = D_{n,p,s}$ can be learned from examples to perfect precision. For our analysis, we require the following fact:

Fact 4 (Fact 3.2 in Ref. [6])**.** *Let $a_1, a_2, \ldots, a_t$ be nonzero integers modulo p, and let $(x_1, x_2, \ldots, x_t) \in \{0,1\}^t$ be sampled uniformly. Then the total variation distance between $\sum_{i=1}^t a_i x_i \mod p$ and U_p, the uniform distribution over $\{0, 1, \ldots, p-1\}$ is at most $\sqrt{p}e^{-t/p^2}$.*

The main contribution of this section is proving the following theorem:

Theorem 5 (Learning algorithm, Theorem 7 in the main text)**.** *There exists a polynomial-time PAC generator learning algorithm for $\mathcal{D}$, that for sufficiently large $p \in O(n^{1/3})$, any $\delta > 0$, and any $s \in \mathbb{F}_p$, outputs a description of a constant-depth quantum circuit whose Born distribution D' satisfies*

$$d_{TV}(D_{n,p,s}, D') = 0 \tag{S9}$$

with probability $1 - \delta$, and which uses $M \in O\left(p^4 \log\left(\frac{p}{\delta}\right)\right)$ examples of $D_{n,p,s}$.

Proof. We are given M examples of the discrete distribution $Q := D_{n,p,s}$ and n, p. To learn Q, our goal is to find s using the M examples and then output a description of the circuit in Fig. 2.a) (main text).

As an intermediate step, let us for now assume that we observe examples from the distribution P defined in Theorem 1. Then Equation (9) (main text) reveals that, given a certain assignment of $Z = (d, x)$, the probability to observe $(d, x, \text{parity}(x))$ in the examples is $\cos^2(-\frac{\pi}{4} + \frac{\pi}{p}(k+s))$, where

$$k = \sum_{i=0}^{n-1} x_i (-1)^{h(d)_i} \mod p. \tag{S10}$$

The strategy for the learning algorithm is essentially to find out the $\cos^2(\cdot)$ values for all $k = 1, \ldots, p$ using M examples. Thus, basically, in the learning algorithm, we build a p-dimensional vector $\vec{V}$ using the M examples, which approximates the p values for $\cos^2(\cdot)$. The $\cos^2(\cdot)$ function is depicted in Fig. 2.c) (main text). From the figure, we can see that if we are able to find the coordinate $p - s \mod p$, it will reveal s to us. Note that the coordinate $p - s$ is an integer. To find this coordinate, we search for the coordinate k^* in $\vec{V}$ where the $\cos^2$ function equals $1/2$ and where it is larger than $1/2$ for $k^* + 1$ (i.e., where $\cos^2$ crosses $1/2$ from below). We can then output our estimate of s by computing $p - k^* \mod p$, which is correct if indeed $k^* = p - s \mod p$. We now describe how we build $\vec{V}$ and how closely it must approximate $\cos^2$ for our learning algorithm to work with high probability.

First, we define a vector $\vec{v}$, which has the property that its expectation value $\mathbb{E}[\vec{v}]$ approximates the p different values for $\cos^2(\cdot)$ as in Fig. 2.c) (main text). We then show how one can build $\vec{V}$ such that it approximates $\mathbb{E}[\vec{v}]$. Define

$$|x^{(i)}| := \sum_{j=0}^{n-1} x_j^{(i)} (-1)^{h(d^{(i)})_j} \tag{S11}$$

for $i = 1, \ldots, M$, where $x^{(i)}$ and $d^{(i)}$ are taken from the i'th example. Then, define the vector

$$\vec{v} := \sum_{i=1}^{M} \mathbb{1}(\text{parity}(x^{(i)}) = Y_{x^{(i)}}) \vec{e}_{|x^{(i)}| \bmod p} p \tag{S12}$$

where $\vec{e}_k$ is the unit vector of dimension p. Since the M examples are drawn randomly, we can interpret $\vec{v}$ as a random variable and analyze its expectation value. Let P_k be the conditional probability of observing the bits (d, x) such that $|x| = k$. For P_k, it follows from Fact 4 that

$$\forall k \in \{0, \ldots, p-1\}, \ \left| P_k - \frac{1}{p} \right| \leq \sqrt{p} \exp\left(-\frac{n-1}{2p^2} \right). \tag{S13}$$

Since the expectation value of $\vec{v}$ is given through

$$\begin{aligned}
\mathbb{E}[\vec{v}]_k &= P_k \times [\cos^2\left(-\frac{\pi}{4} + \frac{\pi}{p}(k+s)\right) \times p + \sin^2\left(-\frac{\pi}{4} + \frac{\pi}{p}(k+s)\right) \times 0] \\
&= P_k \times p \times \cos^2\left(-\frac{\pi}{4} + \frac{\pi}{p}(k+s)\right).
\end{aligned} \tag{S14}$$

Multiplying both sides of Eq. (S13) with p and combining it with Eq. (S14) gives us that the k'th element of $\mathbb{E}[\vec{v}]$ is an $p^{3/2} \exp\left(-\frac{n-1}{2p^2}\right)$-close approximation to $\cos^2\left(-\frac{\pi}{4} + \frac{\pi}{p}(k+s)\right)$.

In the beginning of this proof, we assumed that we observe samples of the distribution P, however, in reality we observe samples from Q. Thus, what we have shown above is that the k'th element of $\mathbb{E}[\vec{v}]_k$ approximates $\cos^2\left(-\frac{\pi}{4} + \frac{\pi}{p}(k+s)\right)$ under P. But in reality we are interested in the approximation under Q. Using Eq. (S8), we find that it holds that

$$|\mathbb{E}_P[\vec{v}]_k - \mathbb{E}_Q[\vec{v}]_k| \leq d_{TV}(P, Q) \times p \leq O(1/n). \tag{S15}$$

And thus the k'th element of $\mathbb{E}_Q[\vec{v}]$ is an $(p^{3/2} \exp\left(-\frac{n-1}{2p^2}\right) + O(1/n))$-close approximation to $\cos^2\left(-\frac{\pi}{4} + \frac{\pi}{p}(k+s)\right)$.

Now, the following lemma shows that that we can efficiently approximate $\mathbb{E}[\vec{v}]_k$ with M examples, for all k.

Lemma 6 (Lemma C.1, page 15 [7])**.** *Let X be a d-dimensional bounded random variable such that $||x||_\infty \leq B$. Given sampling access to X, $\epsilon, \delta > 0$, there exists a classical multivariate mean estimator that returns an ϵ-precise estimate of $\mathbb{E}[X]$ in ℓ_∞-norm, with success probability at least $1 - \delta$ using $O(\frac{B^2}{\epsilon^2} \log(\frac{d}{\delta}))$ samples of X.*

It follows that we can obtain an estimate $\vec{V}$ for $\mathbb{E}_Q[\vec{v}]$, where each entry is ϵ-close, with probability $1-\delta$, using

$$M \in O\left(\frac{p^2}{\epsilon^2}\log\left(\frac{p}{\delta}\right)\right) \tag{S16}$$

many samples. Hence, $\vec{V}_k$ approximates $\cos^2\left(-\frac{\pi}{4}+\frac{\pi}{p}(k+s)\right)$ with error at most $\epsilon + p^{3/2}\exp\left(-\frac{n-1}{2p^2}\right)+O(1/n)$. From the discussions above, we can now give the algorithm to find s.

1. Construct $\vec{V}$ using M samples

$$\vec{V} = \frac{1}{m}\sum_{i=1}^{M}\mathbb{1}(\text{parity}(x^{(i)}) = Y_{x^{(i)}})\vec{e}_{|x^{(i)}| \bmod p}p. \tag{S17}$$

2. Search in $\vec{V}$ for the position k^*, where
   (a) the entry at k^* is τ-close to $\frac{1}{2}$,
   (b) the entry at $k^*+1 \bmod p$ is larger than $\frac{1}{2}+\tau$.

   If no such k^* is found, output *failure*.

3. Output $\tilde{s} = p - k^* \bmod p$.

In order for the algorithm to successfully find s, it is evident from Fig. 2.c) (main text) that τ needs to be smaller than half of the difference between the two consecutive values of $\cos^2\left(-\frac{\pi}{4}+\frac{\pi}{p}(k+s)\right)$ for $k = p-s \bmod p$ and $k = p-s+1 \bmod p$. That is, τ is required to be smaller than

$$\begin{aligned}&\frac{1}{2}\left|\cos^2(-\frac{\pi}{4}) - \cos^2(-\frac{\pi}{4}+\frac{\pi}{p})\right| \\ &= \frac{1}{2}\left|\frac{1}{2} - \cos^2(-\frac{\pi}{4}+\frac{\pi}{p})\right|.\end{aligned} \tag{S18}$$

If that is the case, then our algorithm succeeds. In particular, it suffices that

$$\begin{aligned}\tau &< \frac{1}{2}\left|\frac{1}{2} - \frac{1}{2} + \frac{\pi}{3p}\right| \\ &< \frac{1}{2}\left|\frac{1}{2} - \cos^2(-\frac{\pi}{4}+\frac{\pi}{p})\right|,\end{aligned} \tag{S19}$$

which is true for $p \geq 3$, as proven in Lemma 7 below. And thus we require

$$\tau < \frac{\pi}{6p} \tag{S20}$$

for the algorithm to succeed. It holds that

$$\epsilon + p^{3/2}\exp\left(-\frac{n-1}{2p^2}\right) + O\left(\frac{1}{n}\right) < \frac{\pi}{6p} \tag{S21}$$

asymptotically in p, when $\epsilon = \frac{1}{7p}$ and $n \in \Omega(p^3)$ ($\leftrightarrow p \in O(n^{1/3})$). And thus, for such choices of ϵ, n and using our discussions above, the algorithm to find s succeeds with probability $1-\delta$, when using

$$M \in O\left(\frac{p^2}{\epsilon^2}\log\left(\frac{p}{\delta}\right)\right) \tag{S22}$$

many examples. When we have found s, we can output the circuit in Fig. 2.a) (main text) as the generator. Since the target distribution has exactly been generated by that circuit, the TV distance between the output generator and the target distribution is 0. □

Finally, we show a simple property of the cosine function that is used in the argument above.

Lemma 7 (Property of the cosine function). *For $p \geq 3$, it holds that*

$$\cos^2\left(-\frac{\pi}{4}+\frac{\pi}{p}\right) > \frac{1}{2}+\frac{\pi}{3p}. \tag{S23}$$

Proof. Take $x := 1/p$. We need to show that $f(x) := \cos^2(-\frac{\pi}{4}+\pi x) - \frac{1}{2} - \frac{\pi x}{3} \geq 0$ for $0 \leq x \leq \frac{1}{3}$. It can be easily verified that $f'(0) > 0$ and $f'(\frac{1}{2}) < 0$, and $f''(x) < 0$ for $x \in [0, \frac{1}{2}]$. It follows that f is increasing to a maximum and then decreasing in the interval $[0, \frac{1}{2}]$. Since $f(0) = 0$ and $f(\frac{1}{3}) > 0$, it follows that $f(x) \geq 0$ for $x \in [0, \frac{1}{3}]$. □

---

[1] A. B. Watts and N. Parham, Unconditional quantum advantage for sampling with shallow circuits, (2023), arXiv:2301.00995.

[2] F. Green, S. Homer, C. Moore, and C. Pollett, Counting, fanout, and the complexity of quantum ACC (2001), arXiv:quant-ph/0106017.

[3] M. Reck, A. Zeilinger, H. J. Bernstein, and P. Bertani, Experimental realization of any discrete unitary operator, Phys. Rev. Lett. **73**, 58 (1994).

[4] A. Barenco, C. H. Bennett, R. Cleve, D. P. DiVincenzo, N. Margolus, P. Shor, T. Sleator, J. A. Smolin, and H. Weinfurter, Elementary gates for quantum computation, Phys. Rev. A **52**, 3457 (1995).

[5] E. G. Rieffel and W. H. Polak, *Quantum computing: A gentle introduction* (MIT press, 2011).

[6] E. Viola, The complexity of distributions, SIAM J. Comp. **41**, 191 (2012).

[7] S. Jerbi, A. Cornelissen, M. Ozols, and V. Dunjko, Quantum policy gradient algorithms, in *18th Conference on the Theory of Quantum Computation, Communication and Cryptography (TQC 2023)*, Leibniz International Proceedings in Informatics (LIPIcs), Vol. 266, edited by O. Fawzi and M. Walter (Schloss Dagstuhl – Leibniz-Zentrum für Informatik, Dagstuhl, Germany, 2023) pp. 13:1–13:24.
